# The protective effect of PL 1-3 on D-galactose-induced aging mice

**DOI:** 10.3389/fphar.2023.1304801

**Published:** 2024-01-03

**Authors:** Pengxiao Li, Yazhong Ma, Xiaotong Wang, Xin Li, Xuekun Wang, Jie Yang, Guoyun Liu

**Affiliations:** ^1^ School of Pharmaceutical Sciences, Liaocheng University, Liaocheng, Shandong, China; ^2^ Liaocheng Key Laboratory of Quality Control and Pharmacodynamic Evaluation of Ganoderma Lucidum, Liaocheng University, Liaocheng, Shandong, China

**Keywords:** anti-aging, brain aging, oxidative stress, inflammation, apoptosis, PL 1-3, piperlongumine derivative, D-galactose

## Abstract

The aging population has become an issue that cannot be ignored, and research on aging is receiving increasing attention. PL **1-3** possesses diverse pharmacological properties including anti-oxidative stress, inhibits inflammatory responses and anti-apoptosis. This study showed that PL **1-3** could protect mice, especially the brain, against the aging caused by D-galactose (D-gal). D-gal could cause oxidative stress, inflammation, apoptosis and tissue pathological injury and so on in aging mice. The treatment of PL **1-3** could increase the anti-oxidative stress ability in the serum, liver, kidney and brain of aging mice, via increasing the total antioxidant capacity and the levels of anti-oxidative defense enzymes (superoxide dismutase, glutathione peroxidase, and catalase), and reducing the end product of lipid peroxidation (malondialdehyde). In the brain, in addition to the enhanced anti-oxidative stress via upregulating the level of the nuclear factor erythroid 2-related factor 2 and heme oxygenase 1, PL **1-3** could improve the dysfunction of the cholinergic system via reducing the active of acetylcholinesterase so as to increase the level of acetylcholine, increase the anti-inflammatory and anti-apoptosis activities via downregulating the expressions of pro-inflammatory cytokines (interleukin-6 and tumor necrosis factor-α) and pro-apoptosis proteins (Bcl-2 associated X protein and Caspase-3) in the D-gal-induced aging mice, to enhance the anti-aging ability via upregulating the expression of sirtuin 1 and downregulating the expressions of p53, p21, and p16. Besides, PL **1-3** could reverse the liver, kidney and spleen damages induced by D-gal in aging mice. These results suggested that PL **1-3** may be developed as an anti-aging drug for the prevention and intervention of age-related diseases.

## 1 Introduction

As the population ages, the incidence of age-related ailments such as inflammation, cardiovascular disease, hypertension, diabetes, Alzheimer’s and osteoarthritis has increased significantly (Harshman and Shea, 2016; Wei et al., 2022). The aging population has become an issue that cannot be ignored, and research on aging is receiving increasing attention. Aging is an irreversible physiological process (Esiri, 2007), characterized by the degeneration of function and structure, as well as the decrease of resistance and adaptability. Aging is highly sensitive to many brain changes, which are associated with changes in brain structure and cognitive functions (Oschwald et al., 2019). The pathological characteristics of brain aging are complex, including oxidative stress, inflammation, neuronal degeneration, cell metabolic imbalance and so on (Prolla and Mattson, 2001; Paradies et al., 2011).

D-galactose (D-gal) is an aldohexose that is a commonly used experimental aging inducer. Research has demonstrated that the consumption of D-gal can contribute to the development of aging markers, including advanced glycation end-products (AGEs), the receptor for advanced glycation end-products (RAGEs), telomere shortening, amyloid-β (Aβ), and aging-related pathways such as p53, p21, and p16. p53/p21, and p16/Rb pathways are two important signal pathways that regulate cellular senescence through cell cycle arrest (Herranz and Gil, 2018; Mijit et al., 2020). The arrest of cell cycle is the prerequisite for aging, and the accumulation of p53, p21, and p16 can serve as biomarkers of aging. Furthermore, it can lead to the presence of senescence-associated beta-galactosidase (SA-β-gal) staining positive (Azman and Zakaria, 2019). Accumulating evidence suggests that prolonged exposure to D-gal leads to a reduction in antioxidant enzymes, including catalase (CAT), heme oxygenase 1 (HO-1), superoxide dismutase (SOD), glutathione peroxidase (GPx), and nitric oxide synthase (NOS), resulting in a decrease in overall antioxidant capacity (Dehghani et al., 2019; Wang et al., 2019). Moreover, the protein levels of pro-apoptotic proteins, such as Bcl2-Associated X (Bax), cleaved caspase 3 (active caspase-3) and pro-caspase 3, were increased by D-gal in liver tissue (Shahroudi et al., 2017; Zhang et al., 2019). Overall, chronic D-gal stimulation is considered to successfully mimic the natural aging process by increasing oxidative stress, inflammation, apoptosis and organ injury (Cai et al., 2022). Elevated D-gal level can lead to excessive production of ROS in the body, causing the imbalance of the redox system, leading to oxidative stress, and further inducing aging, especially brain aging. This is because among various organs in the body, the brain is the most susceptible/vulnerable to oxidative stress and relative lack of antioxidant defense (Çakatay, 2010). And the brain aging processes induced by D-gal in mice are similar to those in humans (Ho et al., 2003; El Assar et al., 2013).

**FIGURE 1:**
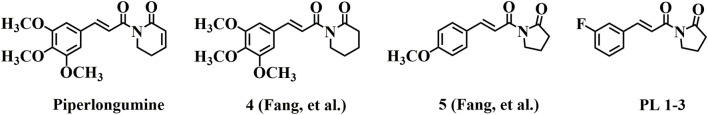
Chemical structures of PL and its active derivatives.

Natural products have historically been extensively researched as a crucial source for discovering new drugs to treat numerous diseases. The lead compounds discovered from natural sources are used as safer and more efficient substitutes for conventional drugs. The Piperlongumine (PL) was originally isolated from fruits of black pepper (Piper nigrum Linn) and long pepper (*Piper longum* L.) (Zhu et al., 2021). Cumulative evidence has suggested that PL and its derivatives elicit a broad spectrum of pharmacological activities, including anti-inflammatory (Sant'Ana et al., 2020), neuroprotective (Bezerra et al., 2013; Jung et al., 2013; Carreiro et al., 2014; Wiemann et al., 2017), antioxidant (Li et al., 2019), anti-cancer (Piska et al., 2018; Tripathi and Biswal, 2020; Sun et al., 2021), antiplatelet aggregation (Wang et al., 2016), anti-diabetes (Rao et al., 2012) and other biological activities. In 2018, Go et al. reported PL treatment has been shown to regulate age-related cognitive decline in age correlates and hippocampal dysfunction in elderly mice (Go et al., 2018). In 2015, Fang et al. reported that two active PL derivatives (**4** and **5**) (Figure 1), owing low cytotoxicity, could protect PC12 cells from oxidative injury via increasing the cellular antioxidant capacity, and could be as potential neuroprotective agents via activating the nuclear factor erythroid 2-related factor 2 (Nrf2) (Peng et al., 2015). Moreover, in our previous study, we found another active derivative PL **1-3** [(*E*)-1-(3-(3-fluorophenyl)acryloyl)pyrrolidin-2-one] (Figure 1) through the structure-activity relationship in the inflammatory cell model (Wang et al., 2021). PL **1-3** owned lower cytotoxicity than PL in Raw264.7 cells and had good oral safety in mice. And it exerted anti-oxidative stress, anti-inflammatory and apoptosis activities, via suppressing the overproductions of reactive nitrogen/oxygen species [NO, malondialdehyde (MDA), and myeloperoxidase (MPO)], pro-inflammatory cytokines [tumor necrosis factor-α (*TNF*-α) and interleukin-1β (*IL-1*β)] and the apoptosis-associated proteins of Bax and Caspase 3 (Wang et al., 2021; Li et al., 2022). However, the anti-aging (especially anti-brain aging) effect of the active derivative PL **1-3** treatment in the D-gal aging model remains unexplored.

In the present study, D-gal-induced subacute aging mouse model was used to detect biomarkers related to aging, inflammation, apoptosis, and oxidative stress-associated in serum, brain tissue and other important organs. We aimed to determine whether active derivatives PL **1-3** had a protective impact on brain injury by D-gal-induced aging in mice and explore its underlying mechanism.

## 2 Results and discussion

### 2.1 PL **1-3** reversed D-gal-induced damages in liver and kidney in aging mice

**FIGURE 2:**
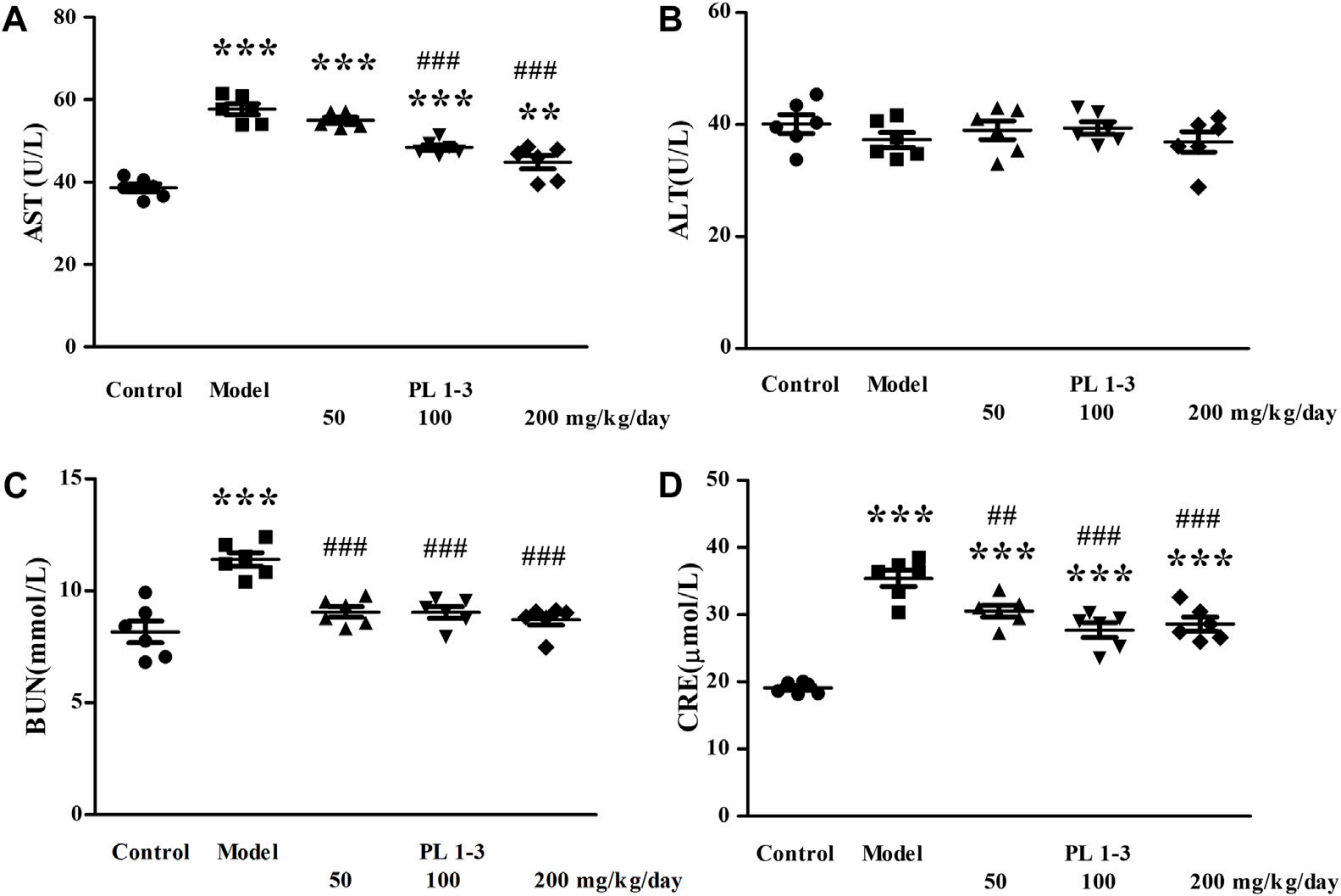
Serum AST **(A)**, ALT**(B)**, BUN **(C)** and CRE **(D)** levels in mice. **p < 0.01, ***p < 0.001, when compared to the control group. ^##^ *p* < 0.01, ^###^ *p* < 0.001, when compared to the model group.

AST, ALT, BUN, and CRE are effective markers for evaluating liver and kidney function injury. As shown in Figure 2, Compared with the control group, the serum AST, BUN, and CRE levels were significantly increased in the model group (Figures 2A, C, D). This suggested that D-gal stimulation caused some damage to the liver and kidney. In contrast, the serum AST, BUN and CRE levels were significantly reduced following PL **1-3** administration compared with those in the model groups.

**FIGURE 3:**
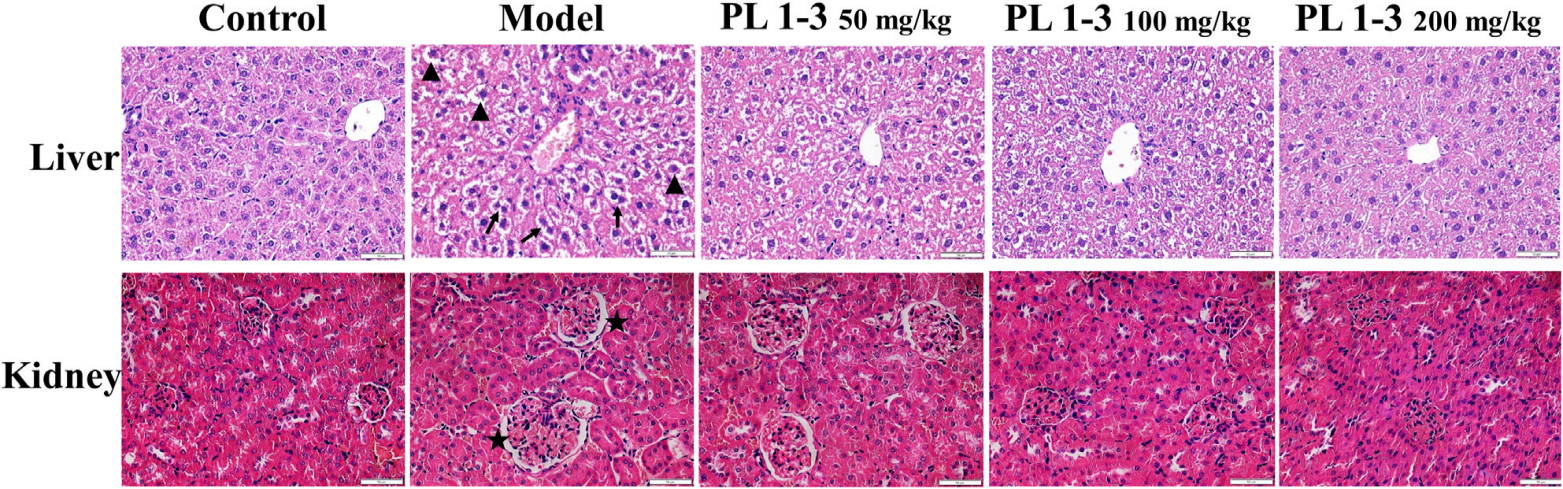
Effects of PL **1-3** in the liver and kidney organ damage in D-gal-induced aging mice. HE staining. (▲, triangle, swollen; →, arrow, binuclear phenomenon; ★, star, an increase in glomerular volume) (Scale bar: liver, 50 μm; kidney, 50 μm).

As depicted in Figure 3, histopathological observations were performed on vital organs including the kidney and liver to support biochemical findings. D-gal treatment induced binucleation of hepatocytes (arrow) and swelling of hepatocytes (triangle) in the liver, and an increase in glomerular volume (star) in the kidney in the model group (Figure 3). However, treatment of PL **1-3** significantly attenuated these organ injuries caused by D-gal. After administration of PL **1-3**, such pathological abnormalities were less frequently observed. The serum biochemical levels and histopathological results suggested that PL **1-3** had potential protective effects on the liver and kidney injury caused by D-Gal.

### 2.2 PL **1-3** protected the serum, liver, kidney and brain against oxidative stress caused by D-Gal in aging mice

Some biomarkers of oxidative stress, the total antioxidant capacity (T-AOC), anti-oxidative defense enzymes (SOD, GPx, and CAT) and an end product of lipid peroxidation (MDA), were measured to evaluate the anti-oxidative stress ability of PL **1-3**.

**FIGURE 4:**
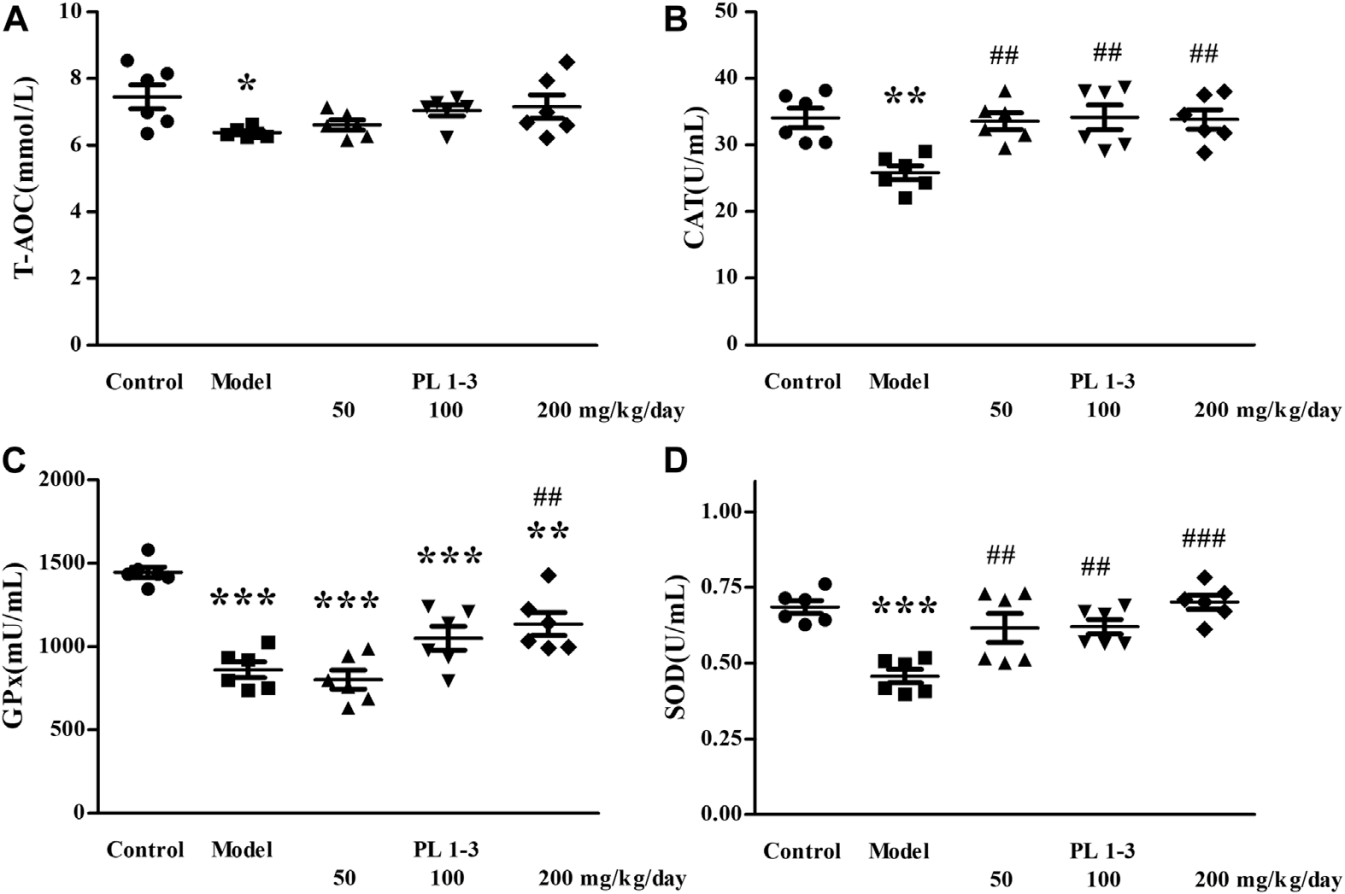
Effects of PL **1-3** on **(A)** T-AOC, **(B)** CAT, **(C)** GPx, and **(D)** SOD levels in the serum of D-gal-induced aging mice. *p < 0.05, **p < 0.01, ***p < 0.001, when compared to the control group. ^##^ *p* < 0.01, ^###^ *p* < 0.001, when compared to the model group.

As shown in Figure 4, T-AOC, CAT, GPx, and SOD levels in the serum of aging mice in the model group were lower than those of normal mice in the control group, which was caused by oxidative stress induced by D-Gal. The treatment of PL **1-3** could weaken or reverse these levels of decrease, thereby enhancing the ability to resist oxidative stress.

**FIGURE 5:**
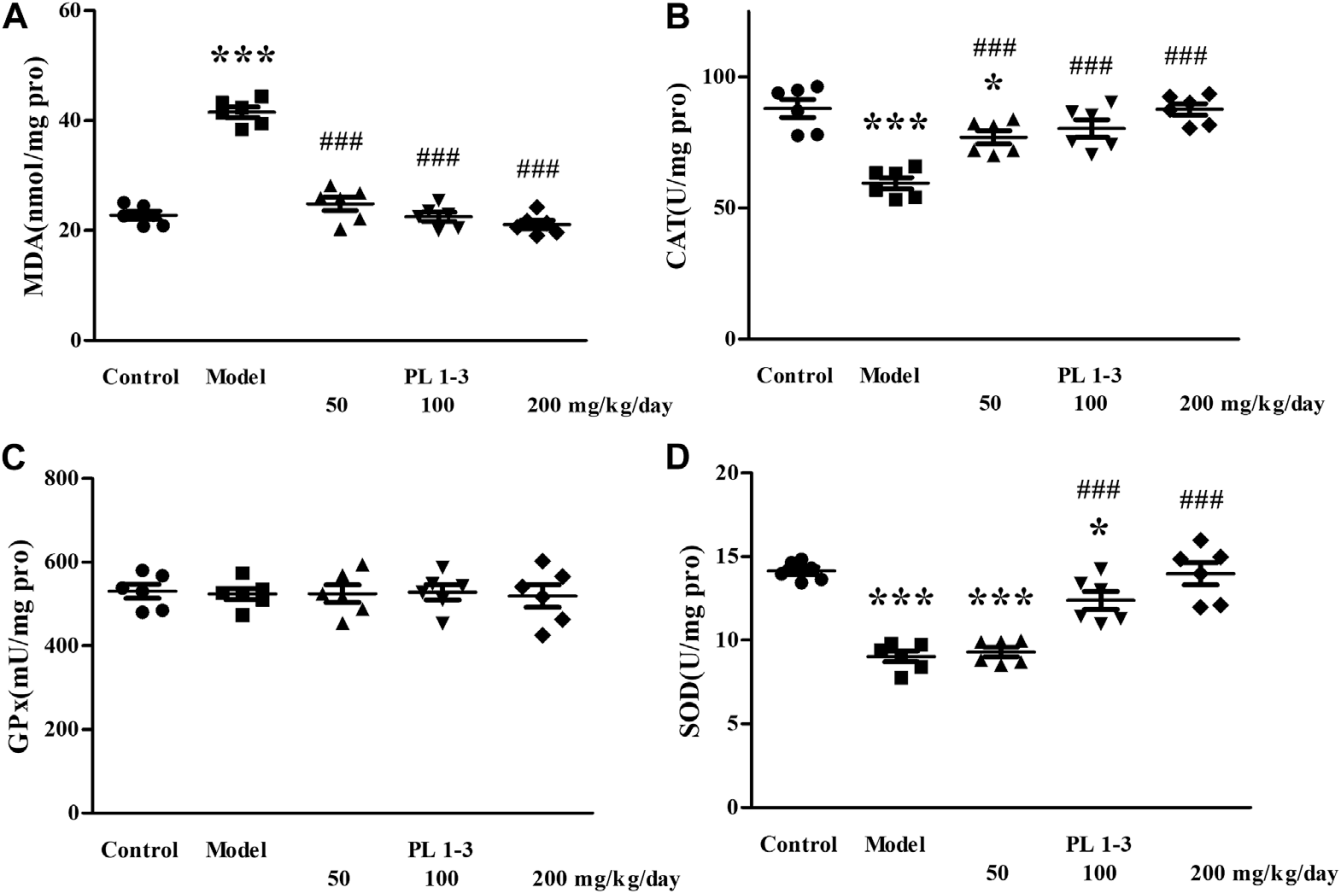
Effects of PL **1-3** on **(A)** MDA, **(B)** CAT, **(C)** GPx, and **(D)** SOD levels in the liver of D-gal-induced aging mice. *p < 0.05, ***p < 0.001, when compared to the control group. ^###^ *p* < 0.001, when compared to the model group.

**FIGURE 6:**
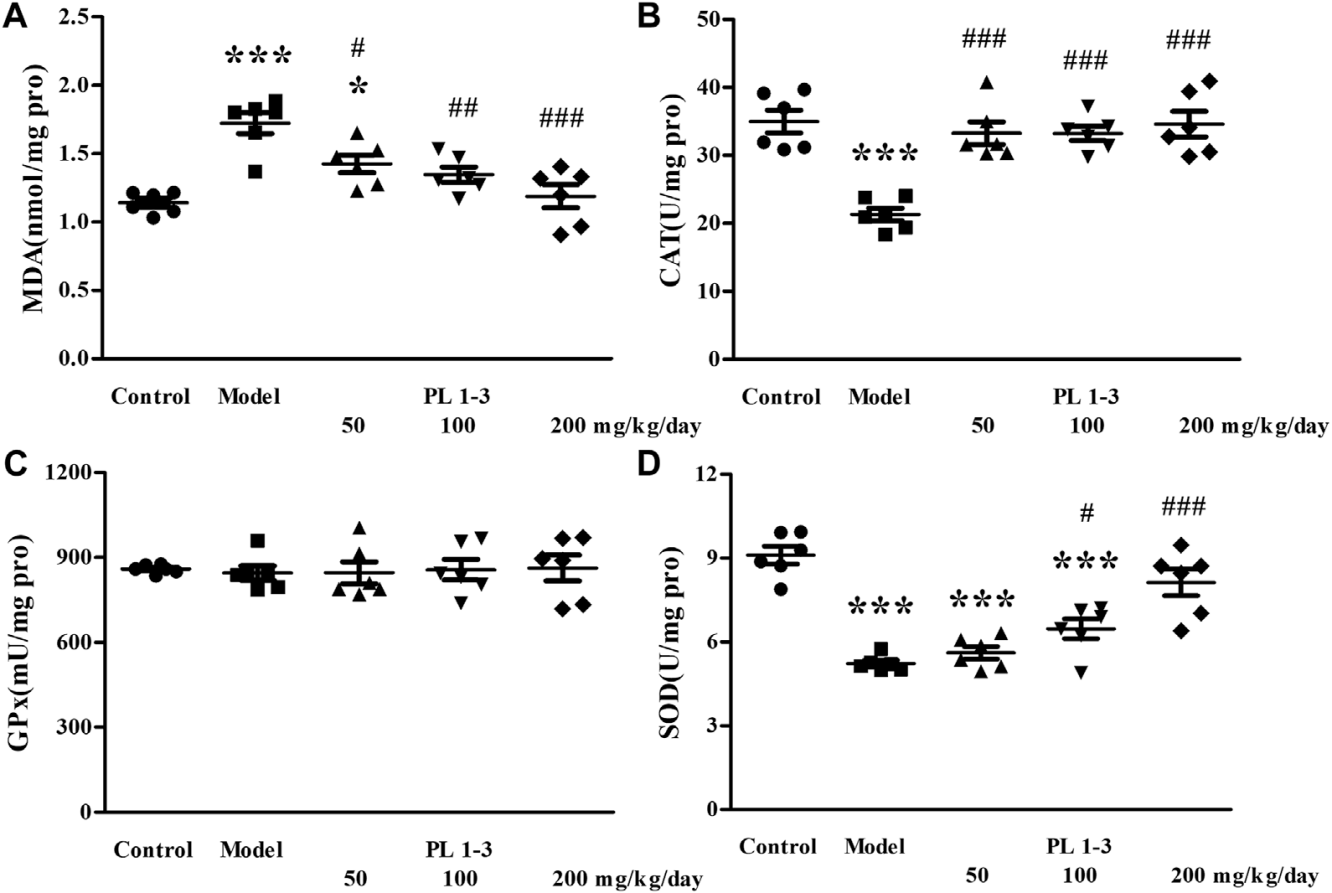
Effects of PL **1-3** on **(A)** MDA, **(B)** CAT, **(C)** GPx, and **(D)** SOD levels in the kidney of D-gal-induced aging mice. *p < 0.05, ***p < 0.001, when compared to the control group. ^#^ *p* < 0.05, ^##^ *p* < 0.01, ^###^ *p* < 0.001, when compared to the model group.

As shown in Figures 5, 6, CAT and SOD levels in the liver and kidney of aging mice in the model group were lower than those of normal mice in the control group, which was caused by oxidative stress induced by D-Gal. PL **1-3** treatment increased those levels.

The MDA level in the liver and kidney of aging mice in the model group was increased by D-Gal, compared with the control group (Figures 5, 6). However, PL **1-3** treatment reduced the increased MDA level. These changes in CAT, SOD and MDA indicated that the liver and kidney were undergoing oxidative stress injury caused by D-gal, but PL **1-3** could increase the anti-oxidative stress capacity of the liver.

**FIGURE 7:**
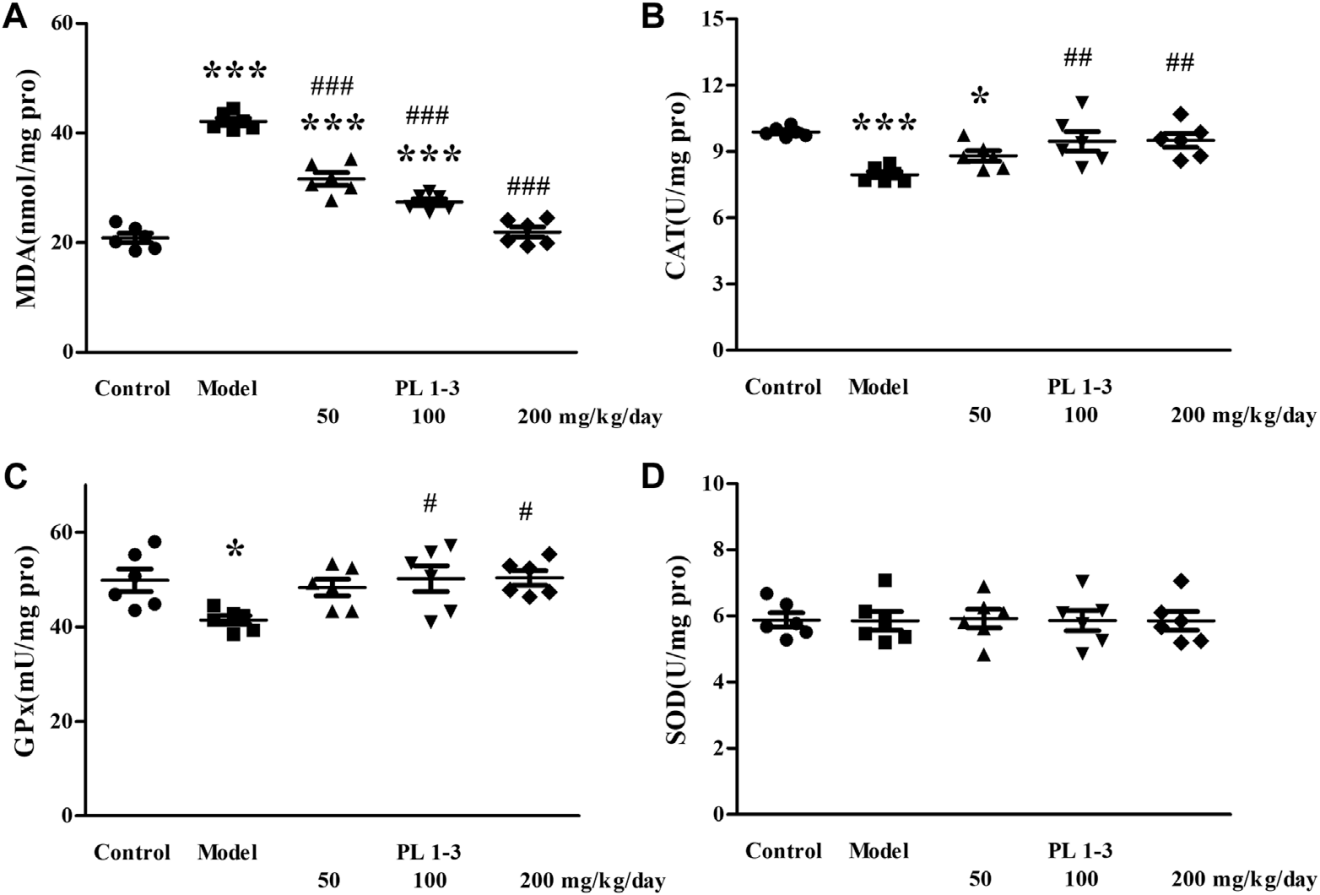
Effect of PL **1-3** on **(A)** T-AOC, **(B)** CAT, **(C)** GPx, and **(D)** SOD levels in the brain of D-gal induced aging mice. *p < 0.05, ***p < 0.001, when compared to the control group. ^#^ *p* < 0.05, ^##^ *p* < 0.01, ^###^ *p* < 0.001, when compared to the model group.

As shown in Figure 7, CAT and GPx levels in the brain of aging mice in the model group were lower than those of normal mice in the control group, which was caused by D-Gal. Similarly, PL **1-3** treatment increased those decreases in CAT and GPx levels caused by D-Gal.

The MDA level in the brain of aging mice in the model group was increased by D-Gal, compared with the control group (Figure 7). The MDA level was decreased by treating with PL **1-3**. These changes in CAT, GPx, and MDA indicated that the brain of aging mice was undergoing oxidative stress injury caused by D-gal, but PL **1-3** could increase the anti-oxidative stress capacity of the brain.

These results related to oxidative stress in serum, liver and brain suggested that PL **1-3** could enhance the anti-oxidative stress ability of mice, and resist the oxidative stress caused by D-gal in aging mice.

### 2.3 PL **1-3** protected the spleen against aging and inflammation caused by D-Gal in mice

The spleen is the largest immune organ within the human body. As age increases, the decline of the immune system weakens the body’s resistance to tumors and pathogens, while increasing the risk of autoimmune diseases and causing chronic inflammatory states.

**FIGURE 8:**
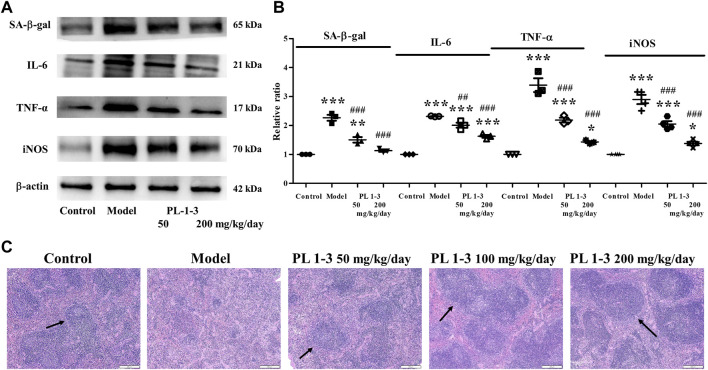
Effects of PL **1-3** on the spleen tissue of D-gal-induced aging mouse. **(A,B)** The expressions of the senescence-associated protein SA-β-gal and the inflammation-associated proteins IL-6, TNF-α, and iNOS; **(C)** HE staining of the spleen tissue. *p < 0.05, **p < 0.01, ***p < 0.001, when compared to the control group. ^##^ *p* < 0.01, ^###^ *p* < 0.001, when compared to the model group. (→, arrow, the white pulp) (Scale bar: spleen, 200 μm).

Interleukin (IL)-6 and TNF-α, two pro-inflammatory cytokines, could promote inflammation and immune regulation. Inducible nitric oxide synthase (iNOS) plays an important role in the production of NO in various diseases such as inflammation, infection and cancer. IL-6, TNF-α, and iNOS were determined using western blot. As shown in Figures 8A, B, the expressions of IL-6, TNF-α and iNOS of the spleen in the aging mice induced by D-gal were higher than those in the control mice. The treatment of PL **1-3** could downregulate the expression of these two pro-inflammatory cytokines. At the same time, the expression of SA-β-gal, a biomarker of aging, was upregulated by D-gal; and was reversed by PL **1-3**. These results suggested that D-gal could cause mice aging and inflammation, and PL **1-3** could protect against inflammation caused by D-gal in the aging mice.

Further, histopathological observations were performed on the spleen (Figure 8). The morphology of normal spleen tissue was normal; however, the white pulp of the spleen (arrow) in aging mice caused by D-gal was significantly reduced in the model group. PL **1-3** treatment improved the pathological condition.

These results suggested that PL **1-3** could protect the spleen against aging, inflammation and histopathological changes caused by D-gal in mice.

### 2.4 PL **1-3** protected the brain against aging by improving the dysfunction of the cholinergic system

The cholinergic system is related to cognitive function. Cholinergic system dysfunction has been observed in several neurodegenerative diseases, such as Alzheimer’s disease, Lewy body dementia and so on (Förstl et al., 2008; Mufson et al., 2008; Ferreira-Vieira et al., 2016). The cholinergic system uses acetylcholine (ACh) as a neurotransmitter. The cholinesterase (AChE) is present in the cholinergic synapse, where it functions to break down ACh. By doing so, it counteracts the excitatory impacts of neurotransmitters on the postsynaptic membrane and guarantees the smooth transmission of neural signals within the body (Zhang et al., 2019). The alterations observed in ACh and AChE levels in the brain serve as crucial markers for investigating the process of brain aging.

**FIGURE 9:**
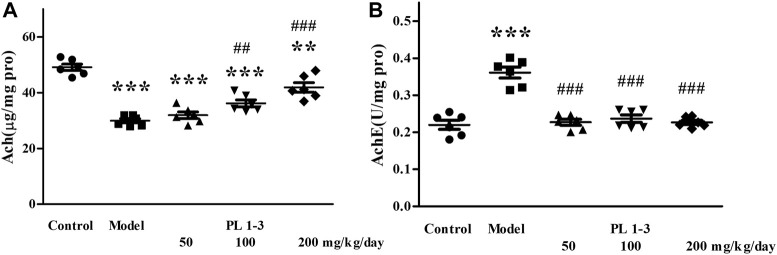
Effects of PL **1-3** on **(A)** ACh and **(B)** AChE levels in the brain of D-gal induced aging mice. **p < 0.01, ***p < 0.001, when compared to the control group. ^##^ *p* < 0.01, ^###^ *p* < 0.001, when compared to the model group.

As shown in Figure 9, long-term injection of D-gal in mice led to the decrease of acetylcholine level and an increase of acetylcholinesterase activity in the model group. However, PL**1-3** increased ACh levels in a dose-dependent manner and significantly inhibited the increase in AChE activity. These results indicate that PL **1-3** could protect the brain against aging induced by D-gal via improving the dysfunction of the cholinergic system.

### 2.5 PL **1-3** improved histopathological alternations of the brain in the aging mice caused by D-gal

**FIGURE 10:**
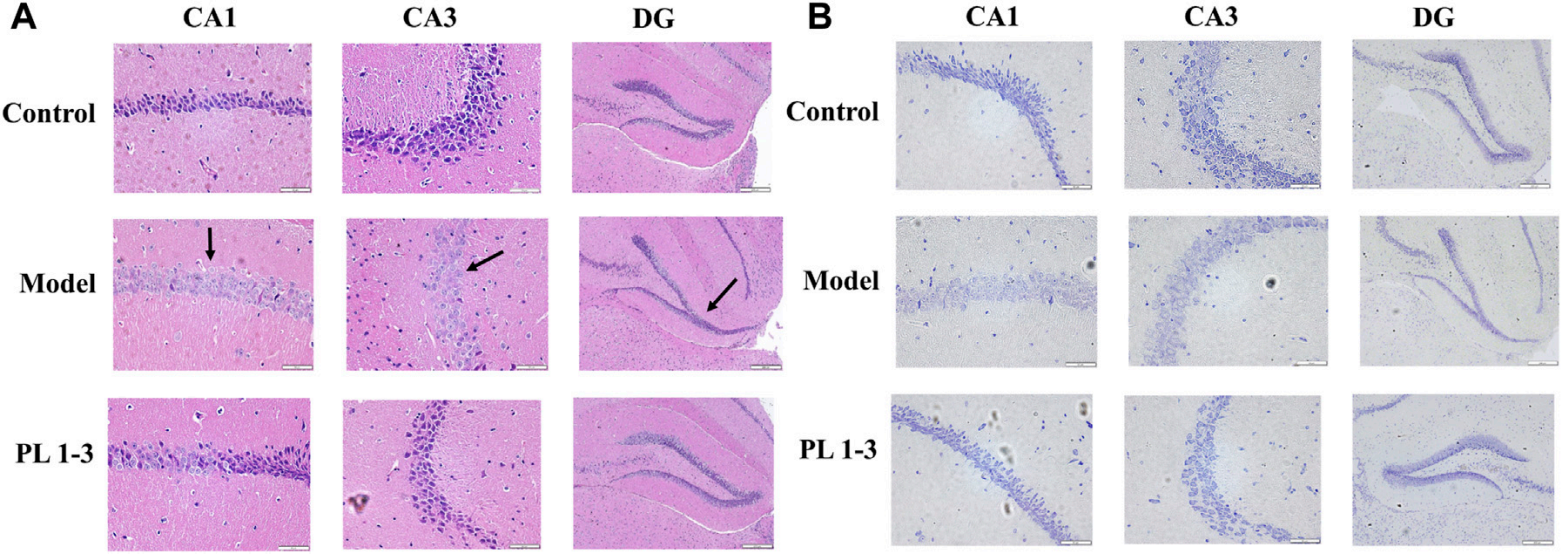
Effects of PL **1-3** on brain injury in hippocampus CA1, CA3 and DG of aging mice caused by D-gal. **(A)** HE staining. **(B)** Nissl staining. (Scale bar: CA1, 50 μm; CA2, 50 μm; DG, 200 μm).

As depicted in Figure 10A, histopathological observations were performed on the brain. There were no pathological alterations in the brain tissues from the control group. In contrast, the morphology of CA1, CA3 and DG in the hippocampus of mice in the model group changed significantly, including loose structure, indistinct cell membranes and changes in the morphology of the DG area (arrow). These results demonstrated that D-gal treatment increased the damage of neurons. After administration of PL **1-3**, such pathological abnormalities were less frequently observed.

Subsequently, Nissl staining was performed to assess the morphology of the hippocampus in mouse brain. Nissl bodies are a type of basophilic material within the cytoplasm which can be stained by Nissl and can be used as status marker for neurons. The morphological changes in hippocampal CA1, CA3, and DG regions were shown in Figure 10B. The number of Nissl bodies in the model group decreased. However, PL **1-3** treatment significantly increased the number of Nissl bodies. HE and Nissl staining showed that PL **1-3** could improve the brain histopathological changes caused by D-gal, improve brain aging caused by D-gal, and inhibit the apoptosis of hippocampal neurons.

### 2.6 PL **1-3** protected the brain against aging by inhibiting oxidative stress, inflammation and apoptosis

During the aging process of the body, the aging of the brain is more sensitive, and the prevention of brain aging is more important. The brain aging is complex, and its pathological features include oxidative stress, inflammation, apoptosis and so on.

#### 2.6.1 Brain aging-associated gene expression in aging mice caused by D-gal

**FIGURE 11:**
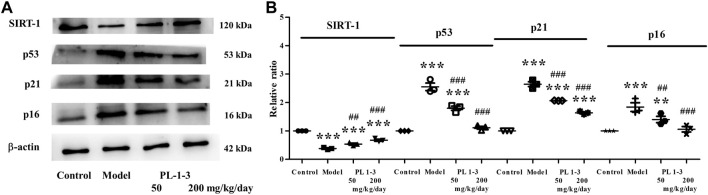
Effects of PL **1-3** on aging (SIRT1, p53, p21, and p16) in the brain tissue. **p < 0.01, ***p < 0.001, when compared to the control group. ^##^ *p* < 0.001, ^###^ *p* < 0.001, when compared to the model group.

As shown in Figure 11, sirtuin 1 (Sirt1), p53, p21, and p16, the senescence-associated proteins, were chosen to assay using western blot. SIRT1 is an important protein related to mammalian aging (Bordone and Guarente, 2005; Hwang et al., 2013). In the process of cellular senescence, there are two signaling pathways-p53/p21 and p16/retinoblastoma protein (pRB), which play crucial roles (Herranz and Gil, 2018; Mijit et al., 2020).

As shown in Figures 11A, B, the expression of SIRT1 was markedly decreased in mice with aging induced by D-gal than that in healthy mice. The treatment of PL **1-3** could upregulate the expression of SIRT1. As shown in Figures 11A, B, in the mice aging model, the expressions of p53, p21, and p16 of the brain were upregulated; however, the treatment of PL **1-3** could reverse these upregulations. These results suggested that, in the mice aging model, the treatment of PL **1-3** could intervene the brain aging and reduce the degree of aging via upregulating SIRT1 and downregulating p53, p21, and p16.

#### 2.6.2 Brain oxidative stress-associated gene expression in aging mice caused by D-gal

Nuclear factor erythroid 2-related factor 2 (Nrf2)/HO-1 signaling pathway is an important anti-oxidant stress pathway in the body, which plays an important role in age-related neurodegenerative diseases and brain aging such as Parkinson’s disease, Alzheimer’s disease and so on.

**FIGURE 12:**
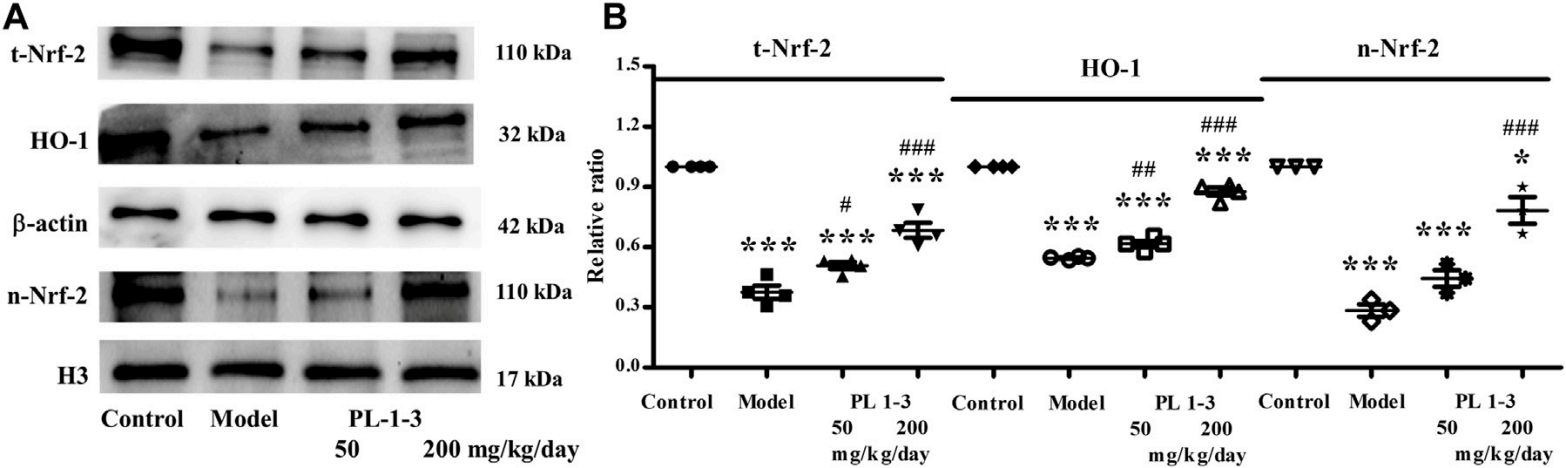
Effects of PL **1-3** on oxidative stress (Nrf2 and HO-1) in the brain tissue. t-Nrf2, total-Nrf2; n-Nrf2, nuclear-Nrf2. *p < 0.05, ***p < 0.001, when compared to the control group. ^#^ *p* < 0.05, ^##^ *p* < 0.01, ^###^ *p* < 0.001, when compared to the model group.

As shown in Figures 12A, B, D-gal downregulated the protein expressions of total-Nrf2, nuclear-Nrf2 and HO-1 of the brain in aging mice, which indicated the anti-oxidative stress ability of aging mice was decreased. The treatment with PL **1-3** upregulated total-Nrf2, nuclear-Nrf2 and HO-1 expressions. These results suggested that PL **1-3** could enhance the antioxidant stress capacity of aging mice via regulating the Nrf2/HO-1 signaling pathway, thereby protecting the brain from aging.

#### 2.6.3 Brain inflammation-associated gene expression in aging mice induced by D-gal

Pro-inflammatory cytokines are a type of cytokines that promote inflammation and immune regulation. IL-6 and TNF-α, two pro-inflammatory cytokines, were chose to assay using western blot.

**FIGURE 13:**
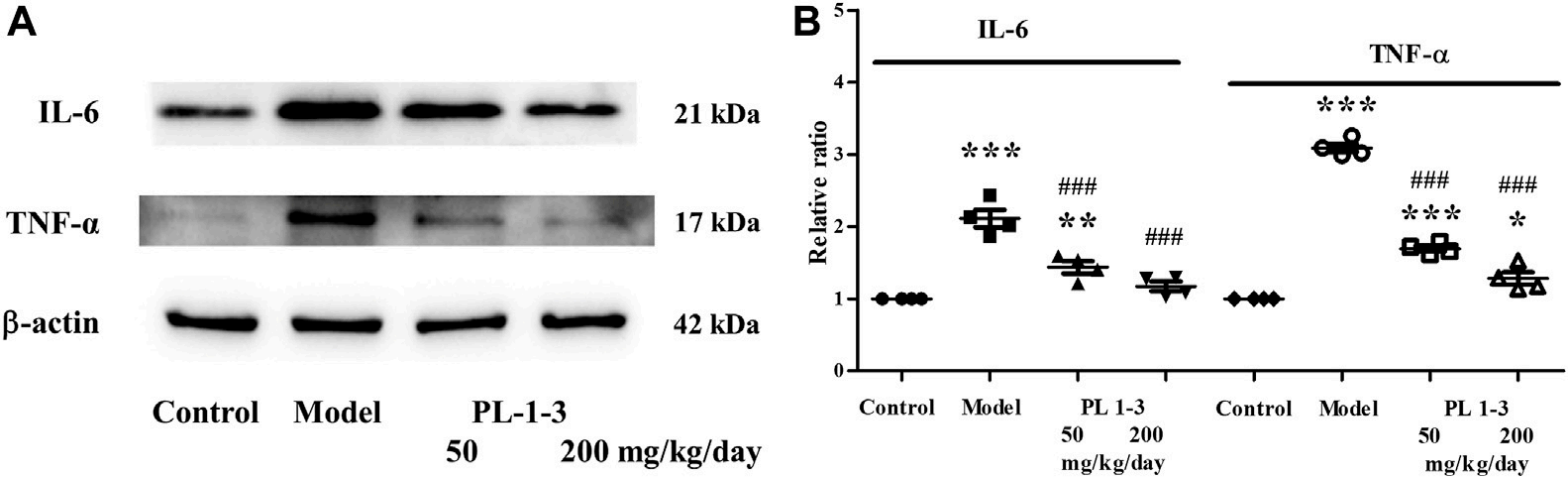
Effects of PL **1-3** on inflammation (IL-6 and TNF-α) in the brain tissue. *p < 0.05, **p < 0.01, ***p < 0.001, when compared to the control group. ^###^ *p* < 0.001, when compared to the model group.

As shown in Figures 13A, B, the expressions of IL-6 and TNF-α of the brain were upregulated in the D-gal-induced aging mice. The treatment with PL **1-3** inhibited the increases of IL-6 and TNF-α in a dose-dependent manner. These results suggested PL **1-3** could resist the brain aging via inhibiting inflammation caused by D-gal.

#### 2.6.4 Brain apoptosis-associated gene expression in aging mice caused by D-gal

The mitochondrial pathway is one of the most important signal transduction pathways of apoptosis, in which the bcl-2 family and caspase family play extremely important roles. Bax belongs to the bcl-2 family and can directly activate the death effector factor caspase-3 or alter cell membrane permeability, causing the release of cytochrome C, activating caspase-3 activity, and initiating caspase cascade reactions.

**FIGURE 14:**
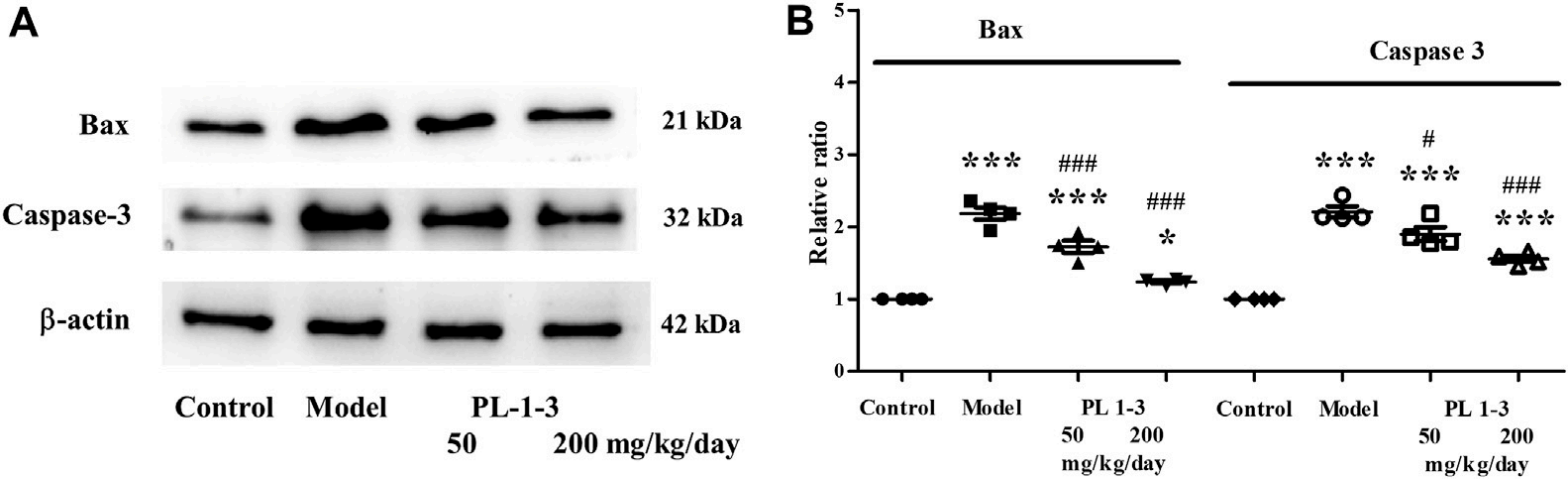
Effects of PL **1-3** on apoptosis (caspase 3 and Bax) in the brain tissue. *p < 0.05, ***p < 0.001, when compared to the control group. ^#^ *p* < 0.05, ^###^ *p* < 0.001, when compared to the model group.

As shown in Figures 14A, B, D-gal caused the upregulation of expressions of the apoptosis-associated proteins Bax and caspase-3 of the brain in aging mice. While, the treatment of PL **1-3** reduced these upregulations in a dose-dependent manner. These results suggested PL **1-3** could inhibit the brain aging of mice caused by D-gal via inhibiting the apoptosis of the brain.

According to research on brain gene expression, PL **1-3** could intervene the brain aging in mice through reducing oxidative stress, inflammation and apoptosis caused by D-gal.

## 3 Conclusion

In summary, this study showed that PL **1-3** could protect mice, especially the brain, against the aging caused by D-gal in mice. The treatment of PL **1-3** could increase the anti-oxidative stress ability (T-AOC, MDA and anti-oxidative enzymes) in the serum, liver, kidney and brain of aging mice. In the brain, in addition to enhancing antioxidant stress, PL **1-3** could also improve the cerebral cholinergic system of the brain by regulating the content of Ach and expression of AchE, increase the anti-apoptosis (Bax and Caspase-3) and anti-inflammatory (IL-6 and TNF-α) activities of aging mice which were induced by D-gal. Besides, PL **1-3** could reverse the liver and kidney damage induced by D-gal in aging mice. These results suggested that PL **1-3** may be developed as an anti-aging drug for the prevention and intervention of age-related diseases.

## 4 Experimental

### 4.1 Materials

Active piperlongumine derivative PL **1-3**, was obtained according to the our previously published procedure (Wang et al., 2021). D-gal was purchased from Amresco Inc. All reagent kits for research were purchased from Applygen Technologies Inc, Nanjing Jiancheng Bioengineering Inc and Beyotime Biotechnology Inc. Antibodies related to western blot experiments were purchased from Wanleibio Inc, Cell Signaling Technology Inc, Beyotime Biotechnology Inc and Santa Cruz Biotechnology Inc. Other reagents used were purchased from Beyotime Biotechnology Inc. Detailed product numbers were described in the experimental steps.

### 4.2 Pre-experiments

According to literature reports, we selected different mice (Kunming mice, male; Kunming mice, female; C57bl/6j mice, male) for preliminary experiments, using different D-gal concentrations and for different experimental times. The content of MDA in the liver was measured and compared with the control group (Table 1). The significant increase in MDA levels served as the indicator for the success of modeling (Yang et al., 2019).

**TABLE 1:** The relative ratio of MDA in the model to the control group.

Group	Mice	D-gal mg/kg/day	Period days	The relative ratio of MDA (Model/Control)
1	Kunming mice, male	100	50	0.98±0.12
2	Kunming mice, female	100	50	1.16±0.14
3	Kunming mice, female	150	80	1.22±0.03
4	Kunming mice, female	300	80	1.29±0.16
5	Kunming mice, female	500	80	1.47±0.07
6	C57bl/6j mice, male	500	70	1.82±0.10

For different gender of Kunming mice, there was the difference in experimental data between male and female mice, and it was difficult to discuss and analyze them together. When the single male C57bl/6j mice were used as the study subjects and continuously intraperitoneally injected 500 mg/kg/day D-gal for 10 weeks, the MDA level of the model group showed a significant increase.

### 4.3 Experimental animals

C57bl/6j mice (7 months old, male, 25 g–35 g) were bought from Pengyue experimental animal company. The National Institute Guide for the Care and Use of Laboratory Animals was the basis for all animal studies. The ethics committee of Liaocheng University gave its approval to the experimental protocols for the utilization of animals. At room temperature, animals were kept in a 12-h light/12-h dark cycle with unrestricted access to sustenance and water.

### 4.4 Brain aging model and animal treatment

After 1 week of acclimatization, mice were randomly divided into 5 groups (*n* = 6 per group), each group was treated with the assigned treatment regimen for 10 weeks: (1) the control group, (2) the model group, (3) the low-dose PL **1-3** group, (4) the middle-dose PL **1-3** group and (5) the high-dose PL **1-3** group. D-gal (500 mg/kg, dissolved in saline), and PL **1-3** (50/100/200 mg/kg, suspended in deionized water). Mice in the control group received intragastric doses of distilled water and intraperitoneal injection with 0.9% normal saline solution. Except for the control group, all four groups of mice were intraperitoneally injected with D-gal once a day. The mice in PL **1-3** groups were administered by gavage once a day. Body weight was monitored every 5 days. The doses of D-gal and PL **1-3** were adjusted according to their body weight. The experiment lasted for 10 weeks.

### 4.5 Determination of blood biochemical indices

Blood samples were taken and centrifuged at 1,000 g at 4°C for 15 min to extract serum. The serum AST and ALT levels were determined by using Aspartate aminotransferase Assay Kit (Applygen Technologies Inc, E2023, China) and Alanine aminotransferase Assay Kit (Applygen Technologies Inc, E2021, China). The serum levels of BUN and CRE were measured by using Urea Assay Kit (Nanjing Jiancheng Bioengineering Institute, C013-1-1, China) and Creatinine Assay kit (Nanjing Jiancheng Bioengineering Institute, C011-2-1, China). Repeat the experiment at least three times.

### 4.6 Oxidative stress level and cholinergic function evaluation

The brain, liver, kidney and spleen tissues were ground in a TissueMaster™ Handheld Homogenizer (Beyotime, E6600, China) with physiological saline. Then, the samples were centrifuged at 12,000 rpm at 4°C for 5 min, the supernatant were stored in new sterile test tube, and the protein concentration were determined using BCA protein assay kit (Beyotime, P0011, China). The CAT, MDA, SOD, T-AOC, and GPx levels in serum, brain, liver, kidney and spleen tissues were measured by chemical kits (Beyotime, China). The AChE and ACh in brain tissues were measured by chemical kits (Nanjing Jiancheng Bioengineering Institute, China). All procedures and concentration calculations in the experiments were carried out in full compliance with the manufacturer’s instructions. Repeat the experiment at least three times.

### 4.7 Histopathological analysis

Liver, kidney and brain tissues were collected and fixed in 4% Paraformaldehyde Fix Solution (Beyotime, P0099, China). The tissue was then dehydrated in a series of alcohols (70%, 80%, 90%, 95%, and 100%), made transparent with xylene, embedded in paraffin, and sectioned. The tissue sections were then dewaxed twice in xylene, eluted with 100%, 90%, 80%, and 70% ethanol and rinsed with water. The sections were then stained with hematoxylin staining solution (Beyotime, C0107, China) for 10 min and washed. Then, the sections were stained with eosin staining solution (Beyotime, C0109, China) for 2 min. After dehydration and transparency again, sections were sealed with the glycerol jelly mounting medium (Beyotime, C0187, China). The sections were viewed under microscopy (Olympus, BX53 + DP80, Japan) for histopathological analysis.

### 4.8 Nissl staining

After dewaxing and eluteing, paraffin sections were stained with Nissl staining solution. (Beyotime, C0117, China) at 37°C for 15 min, and then washed twice with deionized water. The sections were dehydrated twice with 95% alcohol, xylene transparent, and sealed with the glycerol jelly mounting medium (Beyotime, C0187, China). Finally, the changes in Nissl bodies were obtained and analyzed using a microscope (Olympus, BX53 + DP80, Japan).

### 4.9 Western blot analysis

Brain was harvested after treatment, lysed with the Cell lysis buffer for Western and IP (Beyotime, P0013, China) or the frozen sample nuclear protein extraction kit (Kang Lang Biological technology, 202311046, China) added with Protease inhibitor cocktail (Beyotime, P1005, China). Protein samples were separated by SDS-polyacrylamide gel electrophoresis (PAGE) and then transferred to 0.45 µm PVDF membranes (Millipore, IPVH00010, USA). Using 5% non-fat milk blocked the membranes for 1 h and incubated with primary antibodies including Anti-β-actin Rabbit pAb (Wanleibio, WL01372, 1:1,000), Anti-IL-6 Rabbit pAb (Wanleibio, WL02841, 1:1,000), Anti-TNF-α Rabbit pAb (Wanleibio, WL01581, 1:1,000), Bax (Cell Signaling Technology, #2772, 1:1,000), Anti-Caspase-3/Cleaved Caspase-3 Rabbit pAb (Wanleibio, WL02117, 1:1,000), Anti-HO-1/Heme Oxygenase 1 Rabbit pAb (Wanleibio, WL02400, 1:1,000), Nrf2 Rabbit Polyclonal Antibody (Beyotime, AF7623, 1:1,000), Anti-CDKN2A/P16INK4a Rabbit pAb (Wanleibio, WL01418, 1: 1,000), Anti-P21/WAF1 Rabbit pAb (Wanleibio, WL0362, 1: 1,000), Anti-p53 Rabbit pAb (Wanleibio, WL01919, 1: 1,000), Anti-SIRT1 Rabbit pAb (Wanleibio, WL00599, 1:1,000). Membranes were then incubated with the designated antibodies at 4°C overnight, washed, and incubated with secondary antibodies for 1 h at room temperature: HRP-labeled Goat Anti-Mouse IgG (H + L) (Beyotime, A0216, 1:2000) or HRP-labeled Goat Anti-Rabbit IgG (H + L) (Beyotime, A0208, 1:2000). After washing thrice with TBST, protein bands were performed using the Chemi Doc imaging system. (Tanon-4600, China). Repeat the experiment at least three times.

### 4.10 Statistical analysis

The experimental variables were subjected to one-way ANOVA, followed by the Dunnett’s test (compare all columns vs. control column or model column) using GraphPad Prism software. All data were expressed as mean ± SEM. Statistical significance was set at *p* < 0.05.

## Data Availability

The original contributions presented in the study are included in the article, further inquiries can be directed to the corresponding authors.
